# Supplementary material for: A Comprehensive Assessment of Ultraviolet-Radiation-Induced Mutations in Flammulina filiformis Using Whole-Genome Resequencing
Source: J Fungi (Basel). 2024 Mar 20;10(3):228. doi: 10.3390/jof10030228 (PMC10971301; doi:10.3390/jof10030228)
Supplement: Supplementary file 1 [file jof-10-00228-s001.zip › Supplementary Material S8/KEGG annotation/out/64381550635650.os/KO/out_map/map03320.html]

KEGG PATHWAY: PPAR signaling pathway - Reference pathway


|  |  |
| --- | --- |
| **PPAR signaling pathway - Reference pathway** |  |

[
Pathway menu
| Organism menu
| Pathway entry
| Show description
| User data mapping
]

Peroxisome proliferator-activated receptors (PPARs) are nuclear hormone receptors that are activated by fatty acids and their derivatives. PPAR has three subtypes (PPARalpha, beta/delta, and gamma) showing different expression patterns in vertebrates. Each of them is encoded in a separate gene and binds fatty acids and eicosanoids. PPARalpha plays a role in the clearance of circulating or cellular lipids via the regulation of gene expression involved in lipid metabolism in liver and skeletal muscle. PPARbeta/delta is involved in lipid oxidation and cell proliferation. PPARgamma promotes adipocyte differentiation to enhance blood glucose uptake.

|  |  |
| --- | --- |
| Reference pathway | 100% |
